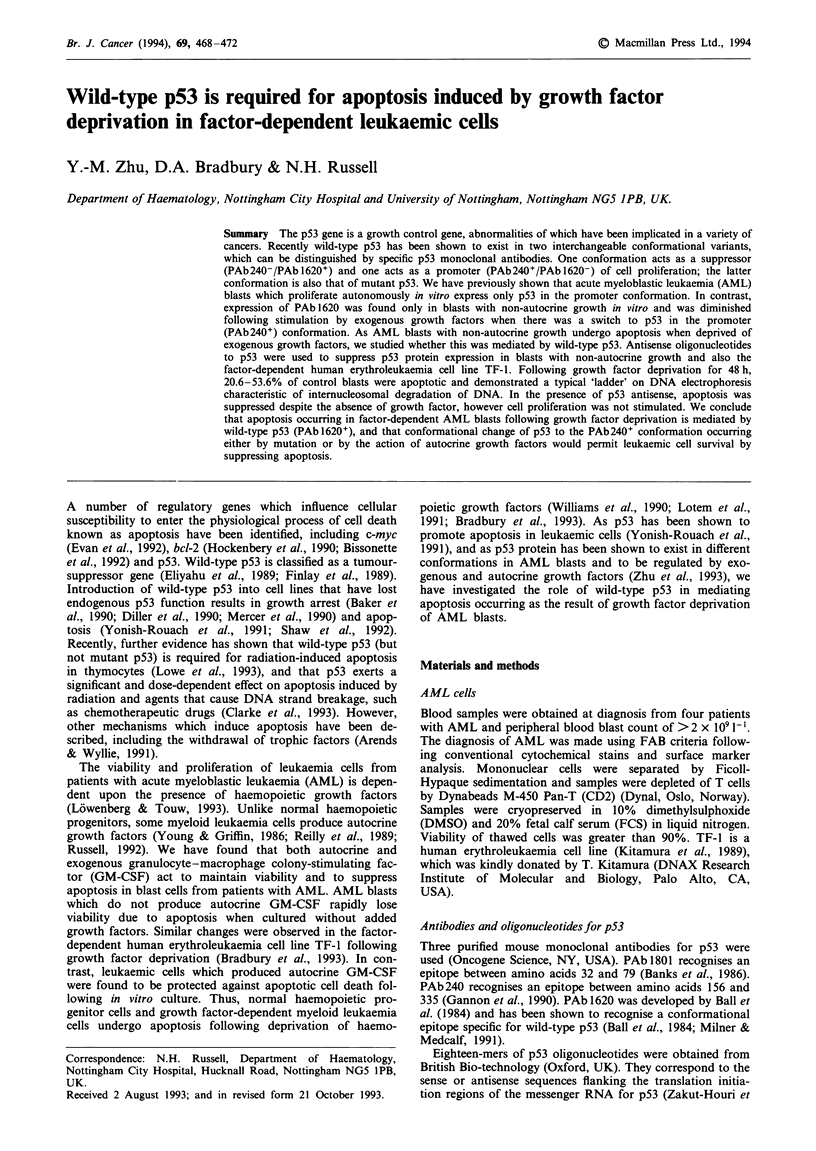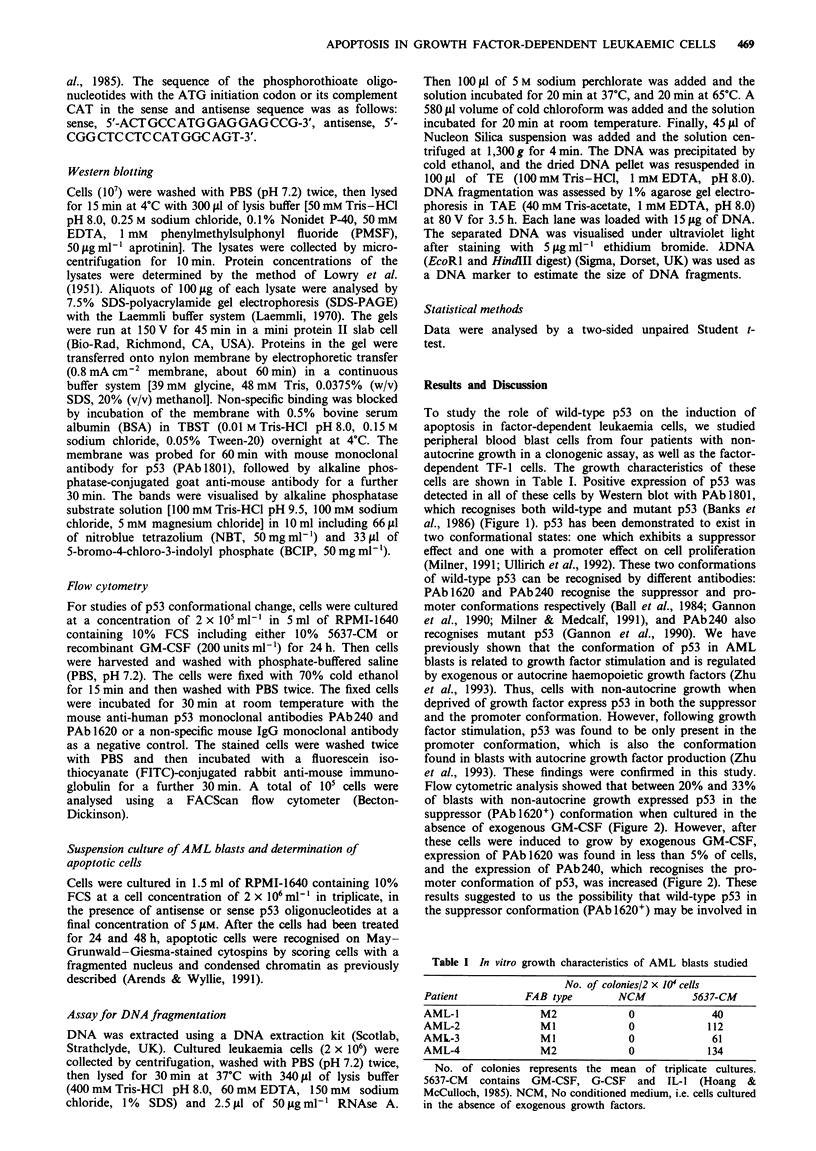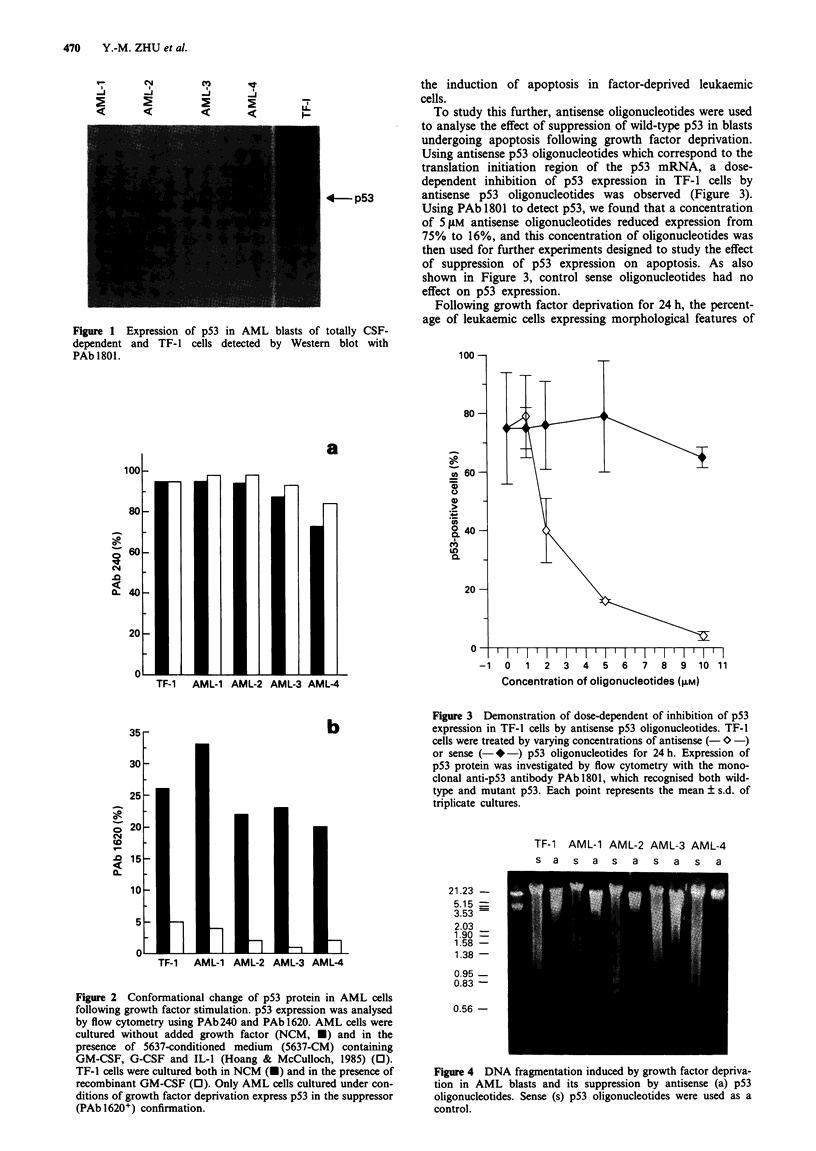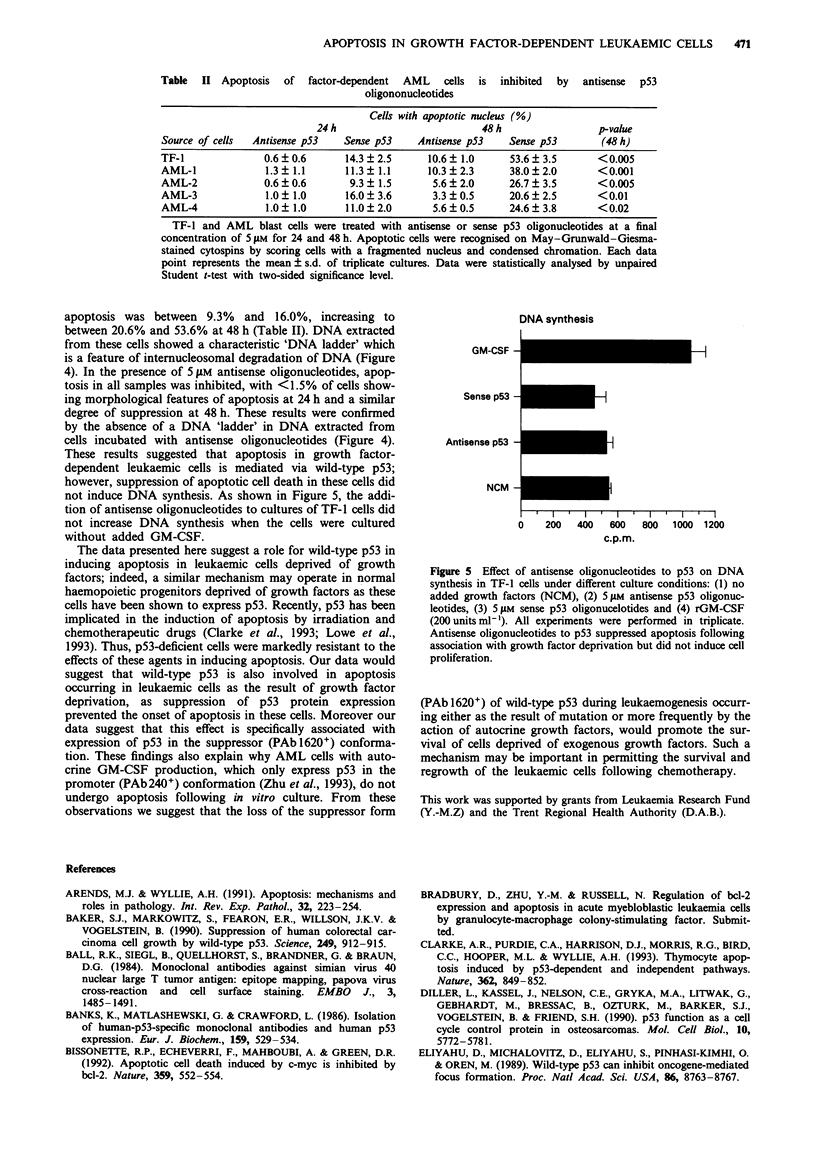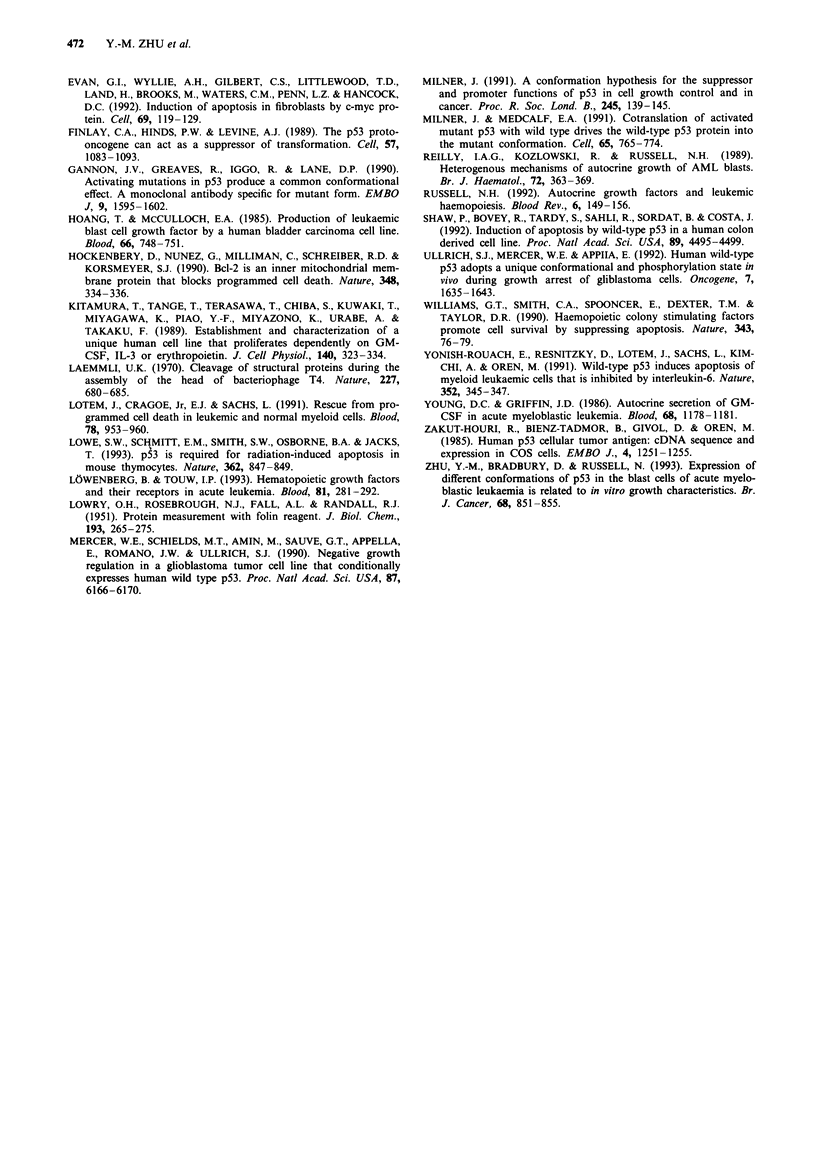# Wild-type p53 is required for apoptosis induced by growth factor deprivation in factor-dependent leukaemic cells.

**DOI:** 10.1038/bjc.1994.85

**Published:** 1994-03

**Authors:** Y. M. Zhu, D. A. Bradbury, N. H. Russell

**Affiliations:** Department of Haematology, Nottingham City Hospital, UK.

## Abstract

**Images:**


*Br. J. Cancer* (1994), **69**, 468–472 © Macmillan Press Ltd., 1994

### Wild-type p53 is required for apoptosis induced by growth factor deprivation in factor-dependent leukaemic cells

Y.-M. Zhu, D.A. Bradbury & N.H. Russell

*Department of Haematology, Nottingham City Hospital and University of Nottingham, Nottingham NG5 1PB, UK.*

**Summary** The p53 gene is a growth control gene, abnormalities of which have been implicated in a variety of cancers. Recently wild-type p53 has been shown to exist in two interchangeable conformational variants, which can be distinguished by specific p53 monoclonal antibodies. One conformation acts as a suppressor (PAb240$^-$/PAb1620$^+$) and one acts as a promoter (PAb240$^+$/PAb1620$^-$) of cell proliferation; the latter conformation is also that of mutant p53. We have previously shown that acute myeloblastic leukaemia (AML) blasts which proliferate autonomously *in vitro* express only p53 in the promoter conformation. In contrast, expression of PAb1620 was found only in blasts with non-autocrine growth *in vitro* and was diminished following stimulation by exogenous growth factors when there was a switch to p53 in the promoter (PAb240$^+$) conformation. As AML blasts with non-autocrine growth undergo apoptosis when deprived of exogenous growth factors, we studied whether this was mediated by wild-type p53. Antisense oligonucleotides to p53 were used to suppress p53 protein expression in blasts with non-autocrine growth and also the factor-dependent human erythroleukaemia cell line TF-1. Following growth factor deprivation for 48 h, 20.6–53.6% of control blasts were apoptotic and demonstrated a typical 'ladder' on DNA electrophoresis characteristic of internucleosomal degradation of DNA. In the presence of p53 antisense, apoptosis was suppressed despite the absence of growth factor, however cell proliferation was not stimulated. We conclude that apoptosis occurring in factor-dependent AML blasts following growth factor deprivation is mediated by wild-type p53 (PAb1620$^+$), and that conformational change of p53 to the PAb240$^+$ conformation occurring either by mutation or by the action of autocrine growth factors would permit leukaemic cell survival by suppressing apoptosis.

A number of regulatory genes which influence cellular susceptibility to enter the physiological process of cell death known as apoptosis have been identified, including c-*myc* (Evan *et al.*, 1992), *bcl*-2 (Hockenbery *et al.*, 1990; Bissonette *et al.*, 1992) and p53. Wild-type p53 is classified as a tumour-suppressor gene (Eliyahu *et al.*, 1989; Finlay *et al.*, 1989). Introduction of wild-type p53 into cell lines that have lost endogenous p53 function results in growth arrest (Baker *et al.*, 1990; Diller *et al.*, 1990; Mercer *et al.*, 1990) and apoptosis (Yonish-Rouach *et al.*, 1991; Shaw *et al.*, 1992). Recently, further evidence has shown that wild-type p53 (but not mutant p53) is required for radiation-induced apoptosis in thymocytes (Lowe *et al.*, 1993), and that p53 exerts a significant and dose-dependent effect on apoptosis induced by radiation and agents that cause DNA strand breakage, such as chemotherapeutic drugs (Clarke *et al.*, 1993). However, other mechanisms which induce apoptosis have been described, including the withdrawal of trophic factors (Arends & Wyllie, 1991).

The viability and proliferation of leukaemia cells from patients with acute myeloblastic leukaemia (AML) is dependent upon the presence of haemopoietic growth factors (Löwenberg & Touw, 1993). Unlike normal haemopoietic progenitors, some myeloid leukaemia cells produce autocrine growth factors (Young & Griffin, 1986; Reilly *et al.*, 1989; Russell, 1992). We have found that both autocrine and exogenous granulocyte–macrophage colony-stimulating factor (GM-CSF) act to maintain viability and to suppress apoptosis in blast cells from patients with AML. AML blasts which do not produce autocrine GM-CSF rapidly lose viability due to apoptosis when cultured without added growth factors. Similar changes were observed in the factor-dependent human erythroleukaemia cell line TF-1 following growth factor deprivation (Bradbury *et al.*, 1993). In contrast, leukaemic cells which produced autocrine GM-CSF were found to be protected against apoptotic cell death following *in vitro* culture. Thus, normal haemopoietic progenitor cells and growth factor-dependent myeloid leukaemia cells undergo apoptosis following deprivation of haemopoietic growth factors (Williams *et al.*, 1990; Lotem *et al.*, 1991; Bradbury *et al.*, 1993). As p53 has been shown to promote apoptosis in leukaemic cells (Yonish-Rouach *et al.*, 1991), and as p53 protein has been shown to exist in different conformations in AML blasts and to be regulated by exogenous and autocrine growth factors (Zhu *et al.*, 1993), we have investigated the role of wild-type p53 in mediating apoptosis occurring as the result of growth factor deprivation of AML blasts.

Correspondence: N.H. Russell, Department of Haematology, Nottingham City Hospital, Hucknall Road, Nottingham NG5 1PB, UK.

Received 2 August 1993; and in revised form 21 October 1993.

### Materials and methods

### *AML cells*

Blood samples were obtained at diagnosis from four patients with AML and peripheral blood blast count of $>2 \times 10^9\,l^{-1}$. The diagnosis of AML was made using FAB criteria following conventional cytochemical stains and surface marker analysis. Mononuclear cells were separated by Ficoll-Hypaque sedimentation and samples were depleted of T cells by Dynabeads M-450 Pan-T (CD2) (Dynal, Oslo, Norway). Samples were cryopreserved in 10% dimethylsulphoxide (DMSO) and 20% fetal calf serum (FCS) in liquid nitrogen. Viability of thawed cells was greater than 90%. TF-1 is a human erythroleukaemia cell line (Kitamura *et al.*, 1989), which was kindly donated by T. Kitamura (DNAX Research Institute of Molecular and Biology, Palo Alto, CA, USA).

### *Antibodies and oligonucleotides for p53*

Three purified mouse monoclonal antibodies for p53 were used (Oncogene Science, NY, USA). PAb1801 recognises an epitope between amino acids 32 and 79 (Banks *et al.*, 1986). PAb240 recognises an epitope between amino acids 156 and 335 (Gannon *et al.*, 1990). PAb1620 was developed by Ball *et al.* (1984) and has been shown to recognise a conformational epitope specific for wild-type p53 (Ball *et al.*, 1984; Milner & Medcalf, 1991).

Eighteen-mers of p53 oligonucleotides were obtained from British Bio-technology (Oxford, UK). They correspond to the sense or antisense sequences flanking the translation initiation regions of the messenger RNA for p53 (Zakut-Houri *et*

*al.*, 1985). The sequence of the phosphorothioate oligonucleotides with the ATG initiation codon or its complement CAT in the sense and antisense sequence was as follows: sense, 5'-ACT GCC ATG GAG GAG CCG-3', antisense, 5'-CGG CTC CTC CAT GGC AGT-3'.

*Western blotting*

Cells ($10^7$) were washed with PBS (pH 7.2) twice, then lysed for 15 min at 4°C with 300 µl of lysis buffer [50 mM Tris–HCl pH 8.0, 0.25 M sodium chloride, 0.1% Nonidet P-40, 50 mM EDTA, 1 mM phenylmethylsulphonyl fluoride (PMSF), 50 µg ml$^{-1}$ aprotinin]. The lysates were collected by microcentrifugation for 10 min. Protein concentrations of the lysates were determined by the method of Lowry *et al.* (1951). Aliquots of 100 µg of each lysate were analysed by 7.5% SDS-polyacrylamide gel electrophoresis (SDS-PAGE) with the Laemmli buffer system (Laemmli, 1970). The gels were run at 150 V for 45 min in a mini protein II slab cell (Bio-Rad, Richmond, CA, USA). Proteins in the gel were transferred onto nylon membrane by electrophoretic transfer (0.8 mA cm$^{-2}$ membrane, about 60 min) in a continuous buffer system [39 mM glycine, 48 mM Tris, 0.0375% (w/v) SDS, 20% (v/v) methanol]. Non-specific binding was blocked by incubation of the membrane with 0.5% bovine serum albumin (BSA) in TBST (0.01 M Tris-HCl pH 8.0, 0.15 M sodium chloride, 0.05% Tween-20) overnight at 4°C. The membrane was probed for 60 min with mouse monoclonal antibody for p53 (PAb 1801), followed by alkaline phosphatase-conjugated goat anti-mouse antibody for a further 30 min. The bands were visualised by alkaline phosphatase substrate solution [100 mM Tris-HCl pH 9.5, 100 mM sodium chloride, 5 mM magnesium chloride] in 10 ml including 66 µl of nitroblue tetrazolium (NBT, 50 mg ml$^{-1}$) and 33 µl of 5-bromo-4-chloro-3-indolyl phosphate (BCIP, 50 mg ml$^{-1}$).

*Flow cytometry*

For studies of p53 conformational change, cells were cultured at a concentration of $2 \times 10^5$ ml$^{-1}$ in 5 ml of RPMI-1640 containing 10% FCS including either 10% 5637-CM or recombinant GM-CSF (200 units ml$^{-1}$) for 24 h. Then cells were harvested and washed with phosphate-buffered saline (PBS, pH 7.2). The cells were fixed with 70% cold ethanol for 15 min and then washed with PBS twice. The fixed cells were incubated for 30 min at room temperature with the mouse anti-human p53 monoclonal antibodies PAb 240 and PAb 1620 or a non-specific mouse IgG monoclonal antibody as a negative control. The stained cells were washed twice with PBS and then incubated with a fluorescein isothiocyanate (FITC)-conjugated rabbit anti-mouse immunoglobulin for a further 30 min. A total of $10^5$ cells were analysed using a FACScan flow cytometer (Becton-Dickinson).

*Suspension culture of AML blasts and determination of apoptotic cells*

Cells were cultured in 1.5 ml of RPMI-1640 containing 10% FCS at a cell concentration of $2 \times 10^6$ ml$^{-1}$ in triplicate, in the presence of antisense or sense p53 oligonucleotides at a final concentration of 5 µM. After the cells had been treated for 24 and 48 h, apoptotic cells were recognised on May–Grunwald–Giesma-stained cytospins by scoring cells with a fragmented nucleus and condensed chromatin as previously described (Arends & Wyllie, 1991).

*Assay for DNA fragmentation*

DNA was extracted using a DNA extraction kit (Scotlab, Strathclyde, UK). Cultured leukaemia cells ($2 \times 10^6$) were collected by centrifugation, washed with PBS (pH 7.2) twice, then lysed for 30 min at 37°C with 340 µl of lysis buffer (400 mM Tris-HCl pH 8.0, 60 mM EDTA, 150 mM sodium chloride, 1% SDS) and 2.5 µl of 50 µg ml$^{-1}$ RNAse A. Then 100 µl of 5 M sodium perchlorate was added and the solution incubated for 20 min at 37°C, and 20 min at 65°C. A 580 µl volume of cold chloroform was added and the solution incubated for 20 min at room temperature. Finally, 45 µl of Nucleon Silica suspension was added and the solution centrifuged at 1,300 *g* for 4 min. The DNA was precipitated by cold ethanol, and the dried DNA pellet was resuspended in 100 µl of TE (100 mM Tris–HCl, 1 mM EDTA, pH 8.0). DNA fragmentation was assessed by 1% agarose gel electrophoresis in TAE (40 mM Tris-acetate, 1 mM EDTA, pH 8.0) at 80 V for 3.5 h. Each lane was loaded with 15 µg of DNA. The separated DNA was visualised under ultraviolet light after staining with 5 µg ml$^{-1}$ ethidium bromide. λDNA (*Eco*R1 and *Hind*III digest) (Sigma, Dorset, UK) was used as a DNA marker to estimate the size of DNA fragments.

*Statistical methods*

Data were analysed by a two-sided unpaired Student *t*-test.

### Results and Discussion

To study the role of wild-type p53 on the induction of apoptosis in factor-dependent leukaemia cells, we studied peripheral blood blast cells from four patients with non-autocrine growth in a clonogenic assay, as well as the factor-dependent TF-1 cells. The growth characteristics of these cells are shown in Table I. Positive expression of p53 was detected in all of these cells by Western blot with PAb 1801, which recognises both wild-type and mutant p53 (Banks *et al.*, 1986) (Figure 1). p53 has been demonstrated to exist in two conformational states: one which exhibits a suppressor effect and one with a promoter effect on cell proliferation (Milner, 1991; Ullirich *et al.*, 1992). These two conformations of wild-type p53 can be recognised by different antibodies: PAb 1620 and PAb 240 recognise the suppressor and promoter conformations respectively (Ball *et al.*, 1984; Gannon *et al.*, 1990; Milner & Medcalf, 1991), and PAb 240 also recognises mutant p53 (Gannon *et al.*, 1990). We have previously shown that the conformation of p53 in AML blasts is related to growth factor stimulation and is regulated by exogenous or autocrine haemopoietic growth factors (Zhu *et al.*, 1993). Thus, cells with non-autocrine growth when deprived of growth factor express p53 in both the suppressor and the promoter conformation. However, following growth factor stimulation, p53 was found to be only present in the promoter conformation, which is also the conformation found in blasts with autocrine growth factor production (Zhu *et al.*, 1993). These findings were confirmed in this study. Flow cytometric analysis showed that between 20% and 33% of blasts with non-autocrine growth expressed p53 in the suppressor (PAb 1620$^+$) conformation when cultured in the absence of exogenous GM-CSF (Figure 2). However, after these cells were induced to grow by exogenous GM-CSF, expression of PAb 1620 was found in less than 5% of cells, and the expression of PAb 240, which recognises the promoter conformation of p53, was increased (Figure 2). These results suggested to us the possibility that wild-type p53 in the suppressor conformation (PAb 1620$^+$) may be involved in

**Table I** *In vitro* growth characteristics of AML blasts studied

| | | *No. of colonies/2 × 10⁴ cells* | |
|---|---|---|---|
| *Patient* | *FAB type* | *NCM* | *5637-CM* |
| AML-1 | M2 | 0 | 40 |
| AML-2 | M1 | 0 | 112 |
| AML-3 | M1 | 0 | 61 |
| AML-4 | M2 | 0 | 134 |

No. of colonies represents the mean of triplicate cultures. 5637-CM contains GM-CSF, G-CSF and IL-1 (Hoang & McCulloch, 1985). NCM, No conditioned medium, i.e. cells cultured in the absence of exogenous growth factors.

**Figure 1** Expression of p53 in AML blasts of totally CSF-dependent and TF-1 cells detected by Western blot with PAb1801.

**Figure 2** Conformational change of p53 protein in AML cells following growth factor stimulation. p53 expression was analysed by flow cytometry using PAb240 and PAb1620. AML cells were cultured without added growth factor (NCM, ■) and in the presence of 5637-conditioned medium (5637-CM) containing GM-CSF, G-CSF and IL-1 (Hoang & McCulloch, 1985) (□). TF-1 cells were cultured both in NCM (■) and in the presence of recombinant GM-CSF (□). Only AML cells cultured under conditions of growth factor deprivation express p53 in the suppressor (PAb1620$^+$) confirmation.

the induction of apoptosis in factor-deprived leukaemic cells.

To study this further, antisense oligonucleotides were used to analyse the effect of suppression of wild-type p53 in blasts undergoing apoptosis following growth factor deprivation. Using antisense p53 oligonucleotides which correspond to the translation initiation region of the p53 mRNA, a dose-dependent inhibition of p53 expression in TF-1 cells by antisense p53 oligonucleotides was observed (Figure 3). Using PAb1801 to detect p53, we found that a concentration of 5 μM antisense oligonucleotides reduced expression from 75% to 16%, and this concentration of oligonucleotides was then used for further experiments designed to study the effect of suppression of p53 expression on apoptosis. As also shown in Figure 3, control sense oligonucleotides had no effect on p53 expression.

Following growth factor deprivation for 24 h, the percentage of leukaemic cells expressing morphological features of

**Figure 3** Demonstration of dose-dependent of inhibition of p53 expression in TF-1 cells by antisense p53 oligonucleotides. TF-1 cells were treated by varying concentrations of antisense (—◇—) or sense (—◆—) p53 oligonucleotides for 24 h. Expression of p53 protein was investigated by flow cytometry with the monoclonal anti-p53 antibody PAb1801, which recognised both wild-type and mutant p53. Each point represents the mean ± s.d. of triplicate cultures.

**Figure 4** DNA fragmentation induced by growth factor deprivation in AML blasts and its suppression by antisense (a) p53 oligonucleotides. Sense (s) p53 oligonucleotides were used as a control.

**Table II** Apoptosis of factor-dependent AML cells is inhibited by antisense p53 oligononucleotides

| | *Cells with apoptotic nucleus (%)* | | | | |
|---|---|---|---|---|---|
| | *24 h* | | *48 h* | | *p-value* |
| *Source of cells* | *Antisense p53* | *Sense p53* | *Antisense p53* | *Sense p53* | *(48 h)* |
| TF-1 | 0.6 ± 0.6 | 14.3 ± 2.5 | 10.6 ± 1.0 | 53.6 ± 3.5 | <0.005 |
| AML-1 | 1.3 ± 1.1 | 11.3 ± 1.1 | 10.3 ± 2.3 | 38.0 ± 2.0 | <0.001 |
| AML-2 | 0.6 ± 0.6 | 9.3 ± 1.5 | 5.6 ± 2.0 | 26.7 ± 3.5 | <0.005 |
| AML-3 | 1.0 ± 1.0 | 16.0 ± 3.6 | 3.3 ± 0.5 | 20.6 ± 2.5 | <0.01 |
| AML-4 | 1.0 ± 1.0 | 11.0 ± 2.0 | 5.6 ± 0.5 | 24.6 ± 3.8 | <0.02 |

TF-1 and AML blast cells were treated with antisense or sense p53 oligonucleotides at a final concentration of 5 µM for 24 and 48 h. Apoptotic cells were recognised on May-Grunwald-Giesma-stained cytospins by scoring cells with a fragmented nucleus and condensed chromation. Each data point represents the mean ± s.d. of triplicate cultures. Data were statistically analysed by unpaired Student *t*-test with two-sided significance level.

apoptosis was between 9.3% and 16.0%, increasing to between 20.6% and 53.6% at 48 h (Table II). DNA extracted from these cells showed a characteristic 'DNA ladder' which is a feature of internucleosomal degradation of DNA (Figure 4). In the presence of 5 µM antisense oligonucleotides, apoptosis in all samples was inhibited, with <1.5% of cells showing morphological features of apoptosis at 24 h and a similar degree of suppression at 48 h. These results were confirmed by the absence of a DNA 'ladder' in DNA extracted from cells incubated with antisense oligonucleotides (Figure 4). These results suggested that apoptosis in growth factor-dependent leukaemic cells is mediated via wild-type p53; however, suppression of apoptotic cell death in these cells did not induce DNA synthesis. As shown in Figure 5, the addition of antisense oligonucleotides to cultures of TF-1 cells did not increase DNA synthesis when the cells were cultured without added GM-CSF.

The data presented here suggest a role for wild-type p53 in inducing apoptosis in leukaemic cells deprived of growth factors; indeed, a similar mechanism may operate in normal haemopoietic progenitors deprived of growth factors as these cells have been shown to express p53. Recently, p53 has been implicated in the induction of apoptosis by irradiation and chemotherapeutic drugs (Clarke *et al.*, 1993; Lowe *et al.*, 1993). Thus, p53-deficient cells were markedly resistant to the effects of these agents in inducing apoptosis. Our data would suggest that wild-type p53 is also involved in apoptosis occurring in leukaemic cells as the result of growth factor deprivation, as suppression of p53 protein expression prevented the onset of apoptosis in these cells. Moreover our data suggest that this effect is specifically associated with expression of p53 in the suppressor (PAb1620$^+$) conformation. These findings also explain why AML cells with autocrine GM-CSF production, which only express p53 in the promoter (PAb240$^+$) conformation (Zhu *et al.*, 1993), do not undergo apoptosis following *in vitro* culture. From these observations we suggest that the loss of the suppressor form (PAb1620$^+$) of wild-type p53 during leukaemogenesis occurring either as the result of mutation or more frequently by the action of autocrine growth factors, would promote the survival of cells deprived of exogenous growth factors. Such a mechanism may be important in permitting the survival and regrowth of the leukaemic cells following chemotherapy.

**Figure 5** Effect of antisense oligonucleotides to p53 on DNA synthesis in TF-1 cells under different culture conditions: (1) no added growth factors (NCM), (2) 5 µM antisense p53 oligonucleotides, (3) 5 µM sense p53 oligonucelotides and (4) rGM-CSF (200 units ml$^{-1}$). All experiments were performed in triplicate. Antisense oligonucleotides to p53 suppressed apoptosis following association with growth factor deprivation but did not induce cell proliferation.

This work was supported by grants from Leukaemia Research Fund (Y.-M.Z) and the Trent Regional Health Authority (D.A.B.).

### References

ARENDS, M.J. & WYLLIE, A.H. (1991). Apoptosis: mechanisms and roles in pathology. *Int. Rev. Exp. Pathol.*, **32,** 223-254.

BAKER, S.J., MARKOWITZ, S., FEARON, E.R., WILLSON, J.K.V. & VOGELSTEIN, B. (1990). Suppression of human colorectal carcinoma cell growth by wild-type p53. *Science*, **249,** 912-915.

BALL, R.K., SIEGL, B., QUELLHORST, S., BRANDNER, G. & BRAUN, D.G. (1984). Monoclonal antibodies against simian virus 40 nuclear large T tumor antigen: epitope mapping, papova virus cross-reaction and cell surface staining. *EMBO J.*, **3,** 1485-1491.

BANKS, K., MATLASHEWSKI, G. & CRAWFORD, L. (1986). Isolation of human-p53-specific monoclonal antibodies and human p53 expression. *Eur. J. Biochem.*, **159,** 529-534.

BISSONETTE, R.P., ECHEVERRI, F., MAHBOUBI, A. & GREEN, D.R. (1992). Apoptotic cell death induced by c-myc is inhibited by bcl-2. *Nature*, **359,** 552-554.

BRADBURY, D., ZHU, Y.-M. & RUSSELL, N. Regulation of bcl-2 expression and apoptosis in acute myebloblastic leukaemia cells by granulocyte-macrophage colony-stimulating factor. Submitted.

CLARKE, A.R., PURDIE, C.A., HARRISON, D.J., MORRIS, R.G., BIRD, C.C., HOOPER, M.L. & WYLLIE, A.H. (1993). Thymocyte apoptosis induced by p53-dependent and independent pathways. *Nature*, **362,** 849-852.

DILLER, L., KASSEL, J., NELSON, C.E., GRYKA, M.A., LITWAK, G., GEBHARDT, M., BRESSAC, B., OZTURK, M., BARKER, S.J., VOGELSTEIN, B. & FRIEND, S.H. (1990). p53 function as a cell cycle control protein in osteosarcomas. *Mol. Cell Biol.*, **10,** 5772-5781.

ELIYAHU, D., MICHALOVITZ, D., ELIYAHU, S., PINHASI-KIMHI, O. & OREN, M. (1989). Wild-type p53 can inhibit oncogene-mediated focus formation. *Proc. Natl Acad. Sci. USA*, **86,** 8763-8767.

EVAN, G.I., WYLLIE, A.H., GILBERT, C.S., LITTLEWOOD, T.D., LAND, H., BROOKS, M., WATERS, C.M., PENN, L.Z. & HANCOCK, D.C. (1992). Induction of apoptosis in fibroblasts by c-myc protein. *Cell*, **69**, 119–129.

FINLAY, C.A., HINDS, P.W. & LEVINE, A.J. (1989). The p53 protooncogene can act as a suppressor of transformation. *Cell*, **57**, 1083–1093.

GANNON, J.V., GREAVES, R., IGGO, R. & LANE, D.P. (1990). Activating mutations in p53 produce a common conformational effect. A monoclonal antibody specific for mutant form. *EMBO J*, **9**, 1595–1602.

HOANG, T. & MCCULLOCH, E.A. (1985). Production of leukaemic blast cell growth factor by a human bladder carcinoma cell line. *Blood*, **66**, 748–751.

HOCKENBERY, D., NUNEZ, G., MILLIMAN, C., SCHREIBER, R.D. & KORSMEYER, S.J. (1990). Bcl-2 is an inner mitochondrial membrane protein that blocks programmed cell death. *Nature*, **348**, 334–336.

KITAMURA, T., TANGE, T., TERASAWA, T., CHIBA, S., KUWAKI, T., MIYAGAWA, K., PIAO, Y.-F., MIYAZONO, K., URABE, A. & TAKAKU, F. (1989). Establishment and characterization of a unique human cell line that proliferates dependently on GM-CSF, IL-3 or erythropoietin. *J. Cell Physiol.*, **140**, 323–334.

LAEMMLI, U.K. (1970). Cleavage of structural proteins during the assembly of the head of bacteriophage T4. *Nature*, **227**, 680–685.

LOTEM, J., CRAGOE, Jr, E.J. & SACHS, L. (1991). Rescue from programmed cell death in leukemic and normal myeloid cells. *Blood*, **78**, 953–960.

LOWE, S.W., SCHMITT, E.M., SMITH, S.W., OSBORNE, B.A. & JACKS, T. (1993). p53 is required for radiation-induced apoptosis in mouse thymocytes. *Nature*, **362**, 847–849.

LÖWENBERG, B. & TOUW, I.P. (1993). Hematopoietic growth factors and their receptors in acute leukemia. *Blood*, **81**, 281–292.

LOWRY, O.H., ROSEBROUGH, N.J., FALL, A.L. & RANDALL, R.J. (1951). Protein measurement with folin reagent. *J. Biol. Chem.*, **193**, 265–275.

MERCER, W.E., SCHIELDS, M.T., AMIN, M., SAUVE, G.T., APPELLA, E., ROMANO, J.W. & ULLRICH, S.J. (1990). Negative growth regulation in a glioblastoma tumor cell line that conditionally expresses human wild type p53. *Proc. Natl Acad. Sci. USA*, **87**, 6166–6170.

MILNER, J. (1991). A conformation hypothesis for the suppressor and promoter functions of p53 in cell growth control and in cancer. *Proc. R. Soc. Lond. B.*, **245**, 139–145.

MILNER, J. & MEDCALF, E.A. (1991). Cotranslation of activated mutant p53 with wild type drives the wild-type p53 protein into the mutant conformation. *Cell*, **65**, 765–774.

REILLY, I.A.G., KOZLOWSKI, R. & RUSSELL, N.H. (1989). Heterogenous mechanisms of autocrine growth of AML blasts. *Br. J. Haematol.*, **72**, 363–369.

RUSSELL, N.H. (1992). Autocrine growth factors and leukemic haemopoiesis. *Blood Rev.*, **6**, 149–156.

SHAW, P., BOVEY, R., TARDY, S., SAHLI, R., SORDAT, B. & COSTA, J. (1992). Induction of apoptosis by wild-type p53 in a human colon derived cell line. *Proc. Natl Acad. Sci. USA*, **89**, 4495–4499.

ULLRICH, S.J., MERCER, W.E. & APPIIA, E. (1992). Human wild-type p53 adopts a unique conformational and phosphorylation state *in vivo* during growth arrest of gliblastoma cells. *Oncogene*, **7**, 1635–1643.

WILLIAMS, G.T., SMITH, C.A., SPOONCER, E., DEXTER, T.M. & TAYLOR, D.R. (1990). Haemopoietic colony stimulating factors promote cell survival by suppressing apoptosis. *Nature*, **343**, 76–79.

YONISH-ROUACH, E., RESNITZKY, D., LOTEM, J., SACHS, L., KIMCHI, A. & OREN, M. (1991). Wild-type p53 induces apoptosis of myeloid leukaemic cells that is inhibited by interleukin-6. *Nature*, **352**, 345–347.

YOUNG, D.C. & GRIFFIN, J.D. (1986). Autocrine secretion of GM-CSF in acute myeloblastic leukemia. *Blood*, **68**, 1178–1181.

ZAKUT-HOURI, R., BIENZ-TADMOR, B., GIVOL, D. & OREN, M. (1985). Human p53 cellular tumor antigen: cDNA sequence and expression in COS cells. *EMBO J.*, **4**, 1251–1255.

ZHU, Y.-M., BRADBURY, D. & RUSSELL, N. (1993). Expression of different conformations of p53 in the blast cells of acute myeloblastic leukaemia is related to *in vitro* growth characteristics. *Br. J. Cancer*, **68**, 851–855.